# Radiological analysis for thoracolumbar disc herniation in spinopelvic sagittal alignment: A retrospective study

**DOI:** 10.1097/MD.0000000000006593

**Published:** 2017-04-07

**Authors:** Tao Wang, Lei Ma, Da-Long Yang, Hui Wang, Di Zhang, Ying-Ze Zhang, Wen-Yuan Ding

**Affiliations:** a Department of Spinal Surgery, The Third Hospital of Hebei Medical University; b Hebei Provincial Key Laboratory of Orthopedic Biomechanics, Shijiazhuang, China.

**Keywords:** lower lumbar disc herniation, spinopelvic sagittal parameters, thoracolumbar disc herniation

## Abstract

A retrospective study aims to explore differences in spinopelvic sagittal alignment between thoracolumbar disc herniation (TLD) and lower lumbar disc herniation (LLD).

A total of 185 patients included 26 with TLD and 129 with LLD and 30 asymptomatic volunteers in normal group (NG). Each individual took full spine X-ray to evaluate pelvic incidence (PI), pelvic tilt (PT), sacral slope (SS), lumbar lordosis (LL), thoracic kyphosis (TK), TK+LL+PI, TK/LL, and sacrum-femoral-pubic symphysis (SFP). The Roussouly classification was used to categorize all subjects according to their sagittal alignment. Spinopelvic parameters and Roussouly classification results were compared between groups.

PI (51.0°), SS (30.5°), and LL (42.0°) in the TLD were significantly higher than those in the LLD (47°, 27°, 33°, respectively). However, TK (30.0°), TK/LL (0.75), and TK+LL+PI (40.0°) in the TLD were significantly lower than these in the LLD (33.0°, 1.07, 47.2°, respectively) and the similar trend between TLD and NG (34.3°, 0.93, 48.5°, respectively). But LL (42.0°) in the TLD was significantly higher than in the NG (35°). Roussouly types among 3 groups were marked differences. The LLD had a higher rate (59.7%) of type II lordosis (flat back), and the TLD had a higher rate (61.5%) of type III lordosis than other groups.

This study implied that patients with TLD have higher LL, lower TK, TK/LL, and TK+LL+PI than LLD patients. We inferred that high LL combined with low TK may be the prospective factors of TLD.

## 1 Introduction

Disc herniation, defined as nucleus pulposus, breaks through the anulus fibrosus causing neurothlipsis. The rate of symptomatic lumbar disc herniation (LDH) is 1% to 3% of general population.^[1]^ The thoracolumbar area were defined as T10-L2,^[2]^ a physiologic junction region, enduring great mobility and amount of weight from ourselves.^[3]^ Some studies reported that disc herniation at the T12-L1, L1-L2, and L2-L3 levels constitute no more than 5% of all LDH.^[3–6]^ No consensus exists on which intervertebral levels constitute the so-called thoracolumbar disc herniation (TLD), while in our studies, we regard disc herniation at T10-T11, T11-T12, T12-L1, L1-2 levels as “thoracolumbar.” TLD may cause back pain, lower limb radiation pain or sensory disturbance, cauda equina, and radiculopathy and even lead to muscle atrophy.^[2,4]^

According to Roussouly type for different degenerative evolution, in younger patients with Type I, characterized by a long thoraco-lumbar kyphosis and a short hyperlordosis, L4-S1 hyperextension might cause L5 spondylolysis. In Type II, flat lordosis, individual have a high risk of LDH. Roussouly just reviewed that Type III was an average shape without characteristics, but not indicated that it caused what kind of disease easily. Type IV was easy to suffer L5 isthmic spondylolysis due to shear forces from a long and curved lumbar.^[7]^

LDH is a common disease among adults with degenerated lumbar intervertebral discs; however, occurrence of TLD is much less frequent in clinic. Although many authors paid more attention on upper and lower LLD, to the best of our knowledge, this is the first study to focus on spinopelvic sagittal parameters for TLD. The purpose of our study is to explore difference in spinopelvic sagittal alignment between TLD and LLD.

## 2 Methods

### 2.1 Subjects

The study was approved by the Institutional Review Board of the Third Hospital of HeBei Medical University before data collection and analysis. The inclusion criteria for patients with LLD or TLD were as follows: patients with nerve compression symptoms such as radioactive leg pain or back pain; 1-level disc herniation was diagnosed by magnetic resonance imaging (MRI); TLD was identified as 1-level disc herniation of T10-T11, T11-T12, T12-L1 or L1-2; LLD was identified as 1-level disc herniation of L4-L5 or L5-S1. Exclusion criteria were that patients with LLD or TLD had a history of any spinal surgery; have spinal deformities (including scoliosis, isthmic spondylolisthesis, irregular endplate, sacralization, or lumbarization); have acute spinal trauma or tumors. Another 30 asymptomatic volunteers as normal group were recruited on the basis of the following inclusion criteria: have no symptom; have no history of back or leg pain and exclusion criteria consisted of patients with history of any spinal surgery, spinal deformities (including scoliosis, isthmic spondylolisthesis, irregular endplate, sacralization, or lumbarization), and have a history of acute spinal trauma or tumors. Finally, a total of 26 patients with TLD including 3 patients with T10/T11disc herniation, 4 patients with T11/T12 disc herniation, 6 patients with T12/L1 disc herniation, and 13 patients with L1/L2 disc herniation and 129 patients with LLD, including 63 patients with L4/5 disc herniation and 66 patients with L5/S1 disc herniation and 30 asymptomatic volunteers from the Third Hospital of HeBei Medicle University were included in this retrospective study, from January 2010 to September 2015.

### 2.2 Radiological assessment

All subjects had a full spine positive and lateral X-ray. Spine surgeons widely received the concept that the sagittal balance as an important factor for managing spinal disease.^[7]^ The importance of pelvic indexes and their relationship (PI = PT+SS) was put forward by Legaye et al^[8,9]^ first. Pelvic incidence (PI) is a fundamental anatomical parameter that is unique for each individual and does not depend on the position or spatial orientation of the pelvis. Two position-dependent variables, sacral slope (SS) and pelvic tilt (PT), determine pelvic orientation in the sagittal plane. PI increases significantly during adolescence, reaching its maximum value in adulthood.^[10]^ The following variables were measured as follows: PI is defined as the angle between the perpendicular to the upper sacral endplate at its midpoint and the line connecting this point to the femoral head axis. PT is defined as the angle between vertical line and line joining hip axis to the center of superior endplate of S1. SS is the defined as the angle between superior endplate of S1 and horizontal line. Lumbar lordosis (LL) is defined as the segmental angle of superior endplate of L1 and superior endplate of S1. Thoracic kyphosis (TK) is the segmental angle of superior endplate of T1 and inferior endplate of T12. TK+LL+PI: We regarded TK and PI as positive(+) and regarded LL as negative. TK/LL: Ratio of TK to LL. Sacrum-femoral-pubic symphysis (SFP) is the angle between line from midpoint of sacrum to femoral and line from femoral to midpoint of pubic symphysis. Body mass index (BMI) is calculated by dividing weight (kg) by the square of height (m).

### 2.3 Statistical analysis

The methods were carried out in accordance with the approved guidelines. Three authors collected and managed all the data of patients according to inclusion and exclusion criteria. In addition, 3 authors were responsible for data analyses. All measurement data are presented as the mean ± SD (standard deviation) when data satisfied criteria for normality with *P* > 0.05. Otherwise, it should be presented as median (interquartile range, IQR). These PI, PT, LL, SS, TK, SFP, TK+LL+PI, TK/LL, BMI and age satisfied criteria for normality and homogeneity of variance; statistical analysis between groups was performed using independent samples *t* test. And count data, such as sex (male/female), Chi-square test was used for data analysis. All statistical analyses were carried out using SPSS, version 21.0 (SPSS Inc., Chicago, IL).

## 3 Results

**Figure 1:**
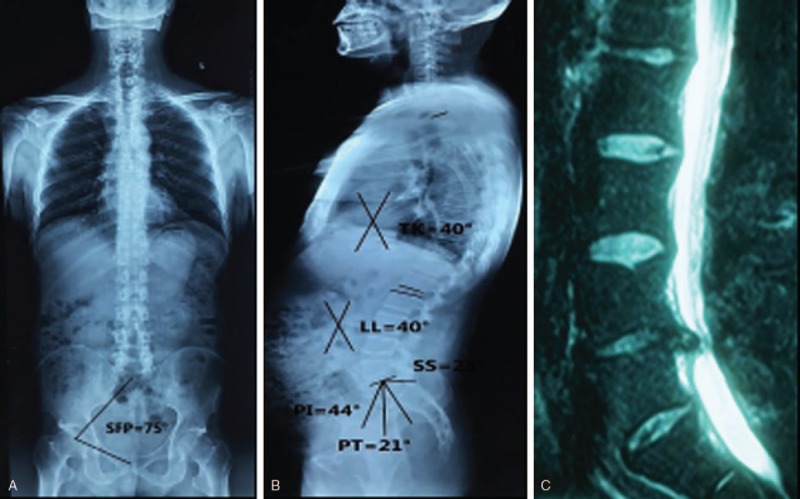
A male, 47 years old. SFP = 75°, PI = 44°, PT = 21°, SS = 23°, LL = 40°, TK = 40°. (A) Positive full spine X-ray; (B) lateral full spine X-ray; (C) lateral lumbar MRI showed disc herniation at L4/L5 level. LL = lumbar lordosis, PI = pelvic incidence, PT = pelvic tilt, SFP = sacrum-femoral-pubic symphysis, SS = sacral slope, TK = thoracic kyphosis.

**Figure 2:**
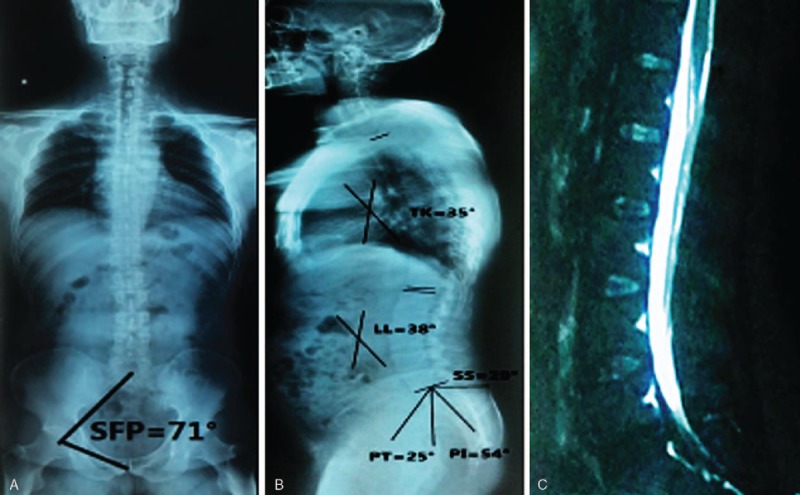
A male, 48 years old. SFP = 71°, PI = 54°, PT = 25°, SS = 29°, LL = 38°, TK = 35°. (A) A positive full spine X-ray; (B) lateral full spine X-ray; (C) lateral lumbar MRI showed no disc herniation. LL = lumbar lordosis, PI = pelvic incidence, PT = pelvic tilt, SFP = sacrum-femoral-pubic symphysis, SS = sacral slope, TK = thoracic kyphosis.

**Figure 3:**
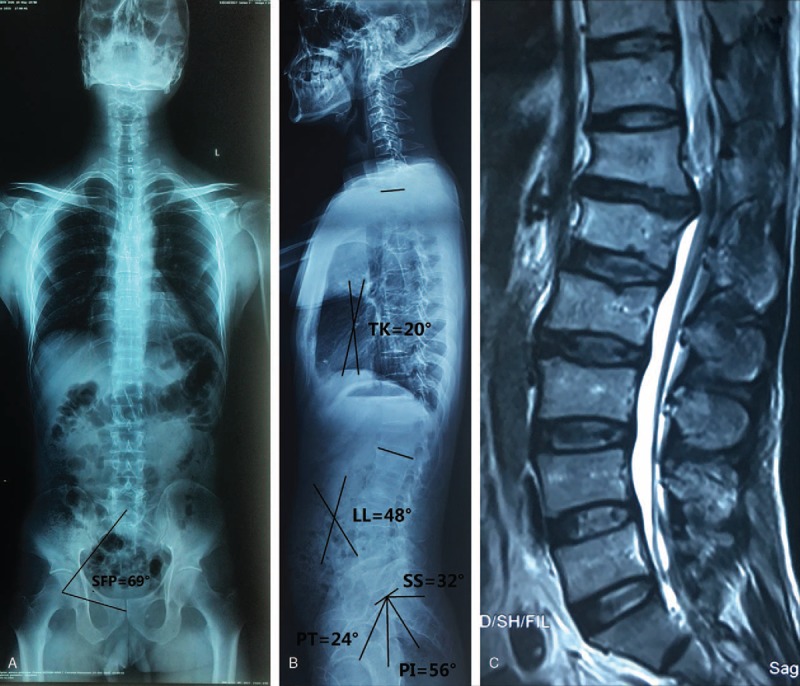
A male, 50 years old. SFP = 69°, PI = 56°, PT = 24°, SS = 32°, LL = 48°, TK = 320°. (A) A positive full spine X-ray; (B) lateral full spine X-ray; (C) lateral lumbar MRI showed disc herniation at T12/L1 level. LL = lumbar lordosis, PI = pelvic incidence, PT = pelvic tilt, SFP = sacrum-femoral-pubic symphysis, SS = sacral slope, TK = thoracic kyphosis.

No significant differences in the age, gender, BMI, SFP, TK, and TK+LL+PI between LLD and NG were noticed. PI (47.0° ± 14.0°), PT (20.0° ± 6.0°), SS (27.0° ± 7.0°), and LL (33.0° ± 6.0°) in the LLD group were significantly lower than those (51.0° ± 6.4°, 21.7° ± 2.8°, 29.4° ± 4.9°, and 35.0° ± 8.0°, respectively) in the NG group; however, TK/LL has an opposite trend (1.07 ± 0.22 vs 0.93 ± 0.13) (Table 1, Figs. 1, 2A–C). As for TLD and NG groups, LL (42.0° ± 5.0°) for TLD was markedly higher than that (35.0° ± 8.0°) for NG; however, TK (30.0° ± 5.4°), TK/LL (0.75 ± 0.16), and TK+LL+PI (40.0° ± 7.0°) for TLD were significantly lower than those (34.3° ± 3.8°, 0.93 ± 0.13, 48.5° ± 6.0°, respectively) for NG (Table 2, Figs. 2 and 3A–C). In TLD and LLD groups, PI (51.0° ± 6.3°), SS (30.5° ± 3.6°), and LL (42.0° ± 5.0°) for TLD were significantly higher than those (47.0° ± 14.0°, 27.0° ± 7.0°, 33.0° ± 6.0°, respectively) for LLD; however, TK (30.0° ± 5.4°), TK/LL (0.75 ± 0.16), and TK+LL+PI (40.0° ± 7.0°) for TLD were significantly lower than those (33.0° ± 9.0°, 0.93 ± 0.13, 47.2° ± 8.8°, respectively) for LLD (Table 3, Figs. 1 and 3A–C).

**Table 1:**
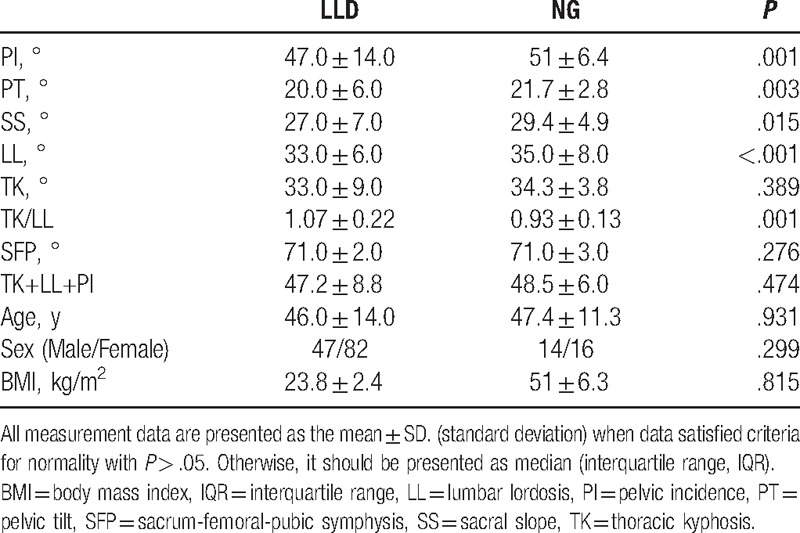
Comparison of spinopelvic parameters between lower lumbar disc herniation (LLD) and normal group (NG).

**Table 2:**
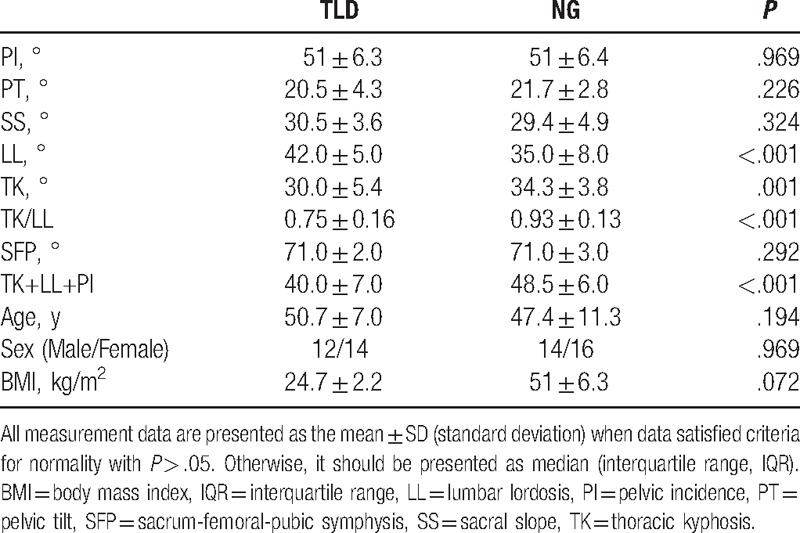
Comparsion of spinopelvic parameters between thoracolumbar disc herniation (TLD) and normal group (NG).

**Table 3:**
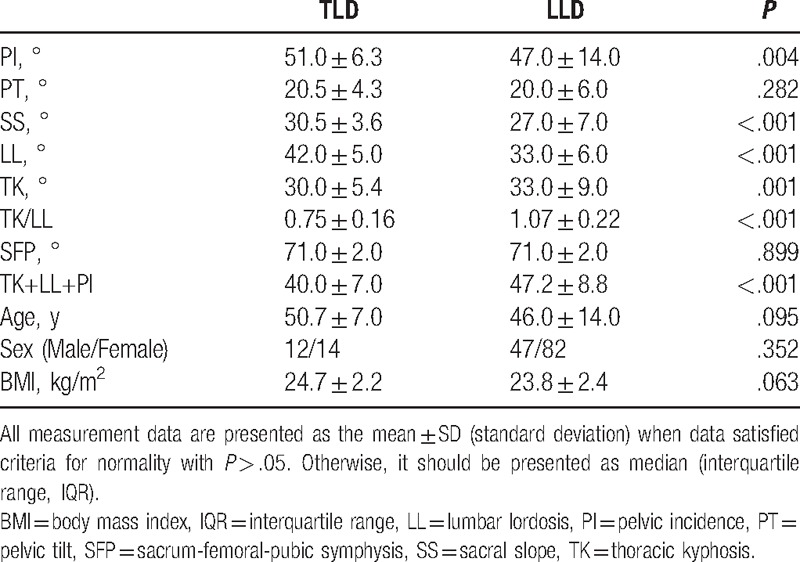
Comparsion of spinopelvic parameters between thoracolumbar disc herniation (TLD) and lower lumbar disc herniation (LLD).

There were significant differences among the 3 groups in Roussouly types. The LLD had higher proportion (59.7%) of type II lordosis (flat back), and the TLD had a higher proportion (61.5%) of type III lordosis than the other groups (Table 4).

**Table 4:**
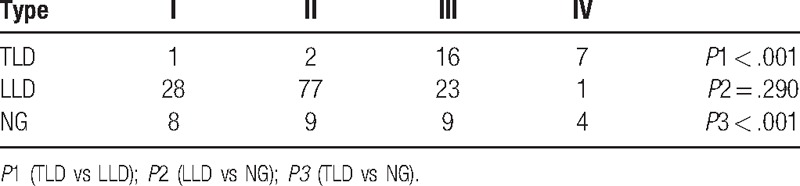
Roussouly types among 3 groups (Type I is a long thoracolumbar kyphosis and a short hyperlordosis: discopathies in the TL area; Type II is a flat lordosis; Type III has an average shape without characteristics for a specific degeneration of the spine; Type IV is a long and curved lumbar spine).

## 4 Discussion

Spinopelvic sagittal alignment accompanied with biomechanical changes has been demonstrated by previous studies in the pathogenesis and development of lumbar degenerative diseases.^[11–20]^ Several studies compared LDH patients with the asymptomatic volunteers in the sagittal spinopelvic parameters, suggesting that patients with LDH was characterized by a straight spine (lower LL and TK), vertical sacrum.^[12–14,16,21,22]^ Nevertheless, few studies reported on spinopelvic parameters for TLD. The aim of this study is to explore the sagittal spinopelvic parameters for TLD and compare sagittal spinopelvic parameters between TLD and LLD.

Our results showed that PI, SS, and LL in TLD group were significantly higher than those in LLD group, but TK, TK/LL, and TK+LL+PI in TLD group were significantly lower than those in LLD group. LL for TLD was markedly higher than that for NG. However, the TK, TK/LL, and TK+LL+PI for TLD were significantly lower than those for NG. Patients with TLD had a higher proportion (61.5%) of type III lordosis; however, LLD patients had higher proportion (59.7%) of type II lordosis.

In our study, PI (51.0°), SS (30.5°), and LL (42.0°) for TLD were significantly higher than PI (47.0°), SS (27.0°), and LL (33.0°) for LLD; however, TK (30.0°) and TK+LL+PI (40.0°) for TLD were significantly lower than TK (33.0°) and TK+LL+PI (47.2°) for LLD. The data indicated that patients with TLD had smaller curvatures in thoracic segment and larger curvatures in lumbar segment than LLD patients. Compared with lower lumbar, thoracolumbar endured force from our movement, such as weight-bearing activities more concentrated with lower TK and larger LL. Besides, the thoracolumbar area was a physiologic junction region, enduring great mobility and amount of weight from ourselves.^[2]^ Above all, greater compressive force speed up the degeneration of the disc, which increases the risk of TLD.

The same has been reported by previous studies^[23]^: PI (47.0°), PT (20.0°), SS (27.0°), and LL (33.0°) for LLD were significantly lower than PI (51.0°), PT (21.7°), SS (29.4°), and LL (35.0°) for NG. The small SS and PI found in LLD suggest that the spatial orientation of the sacrum is more vertical than in the NG. The small degree of LL, the lumbar angle, and the amplitude of the curvatures, together with the high value of the inclination of the spine, show that the sagittal shape of the spine in these patients is straight with only small curvatures.^[8,12–14,16,21,22]^ In this situation, the compressive force component of gravity increases and then it can accelerate the degeneration of the disc. This increase of the compressive forces may lead to herniation.^[20–22]^

Patients with TLD had markedly higher LL (42.5°) than that (39.0°) for asymptomatic volunteers; however, TK (30.0°) and TK+LL+PI (40.0°) for TLD were significantly lower than TK (34.3°) and TK+LL+PI (48.5°) for NG. Similarly previous stated, the results certified the smaller curvatures in thoracic segment and larger curvatures in lumbar segment in TLD than these in NG. Due to more concentrated compressive force and physiologic junction region, thoracolumbar disc degeneration would be faster than LLD and NG.

Ratio of PT to SS (PT/SS) was used by Mac-Thiong et al^[23]^ to indicate the positioning of the pelvis and was considered >1 as an ideal alignment without retroverting compensation. We also performed the similar exploration and our data showed that patients with TLD had lower TK/LL than LLD patients (0.75, 1.07, respectively) and the same trend between NG and LLD (0.93, 1.07, respectively), suggesting that a small thoracic curve and a large lumbar curve were the characteristic for patients with TLD. As we know, thoracolumbar area, physiological curvature change area, is the concentration of biological stress. Patients, with small thoracic curve and large lumbar curve, are more likely to have degeneration of thoracolumbar disc than the patient with large thoracic curve and small lumbar curve. The spine with small TK and large LL leads to more stress focusing on thoracolumbar area, which speed up disc degeneration. However, spine with large TK and small LL have smooth curve, which make stress dispersion out of thoracolumbar area relatively.

Type I has short LL and long kyphosis along with an extension on the thoracolumbar area, which is a no harmonious back with thoracolumbar kyphosis and short hyperlordosis; Type II is a flat back longer like a straight line; Type III has a harmonious regular back with well balanced between its 2 arches; Type IV is a harmonious hypercurved back with highly increased in angle distal arch, number of vertebrae, and length and curvature of LL increase.^[7]^ According to Roussouly types,^[24]^ there were significant differences among the 3 groups. The TLD had a higher proportion (61.5%) of type III lordosis than the other groups, and the LLD had a significantly higher proportion (59.7%) of type II lordosis. Bae et al^[25]^ reported that LLD had a significantly higher proportion (62.6 %) of type II lordosis and the upper lumbar disc herniation had a significantly higher proportion (33.3 %) of type I lordosis. The above results are consistent with that of Roussouly.^[7,17]^ Roussouly reported that Type III, without characteristics, was an average shape, but not indicated that it caused what kind of disease easily. Roussouly^[7]^ showed the 4 types for different degenerative evolution, compared with Type II, Type III had a higher PI and LL, but had a lower TK, which were consistent with our results.

The present study has several limitations. First, a small sample size may limit the accuracy of this study due to relatively low incidence of TLD; second, this is just a retrospective study and we also need a prospective study to observe the change of sagittal spinopelvic for development for TLD; third, these data just form single-center and we need multicenter data in further study. However, to the best of our knowledge, this is the first study report on sagittal spinopelvic parameters for TLD and compare it with lower disc herniation and normal group.

In conclusion, this is the first study to compare spinopelvic parameters between TLD and LLD. We found that patients with TLD had larger LL and smaller TK than patients with LLD, causing stress concentration at thoracolumbar area. So, we inferred that high LL, low TK, TK/LL, and TK+LL+PI may be a risk factor for TLD.
